# Bipartite Genomes in *Enterobacterales*: Independent Origins of Chromids, Elevated Openness and Donors of Horizontally Transferred Genes

**DOI:** 10.3390/ijms24054292

**Published:** 2023-02-21

**Authors:** Cecilie Bækkedal Sonnenberg, Peik Haugen

**Affiliations:** Department of Chemistry, The Center for Bioinformatics (SfB), Faculty of Science and Technology, UiT The Arctic University of Norway, N-9037 Tromsø, Norway

**Keywords:** *Vibrionaceae*, Pseudoalteromonas, multipartite, bipartite, pangenome, horizontal gene transfer, codon usage bias, chromid

## Abstract

Multipartite bacteria have one chromosome and one or more chromid. Chromids are believed to have properties that enhance genomic flexibility, making them a favored integration site for new genes. However, the mechanism by which chromosomes and chromids jointly contribute to this flexibility is not clear. To shed light on this, we analyzed the openness of chromosomes and chromids of the two bacteria, *Vibrio* and *Pseudoalteromonas*, both which belong to the *Enterobacterales* order of *Gammaproteobacteria*, and compared the genomic openness with that of monopartite genomes in the same order. We applied pangenome analysis, codon usage analysis and the HGTector software to detect horizontally transferred genes. Our findings suggest that the chromids of *Vibrio* and *Pseudoalteromonas* originated from two separate plasmid acquisition events. Bipartite genomes were found to be more open compared to monopartite. We found that the shell and cloud pangene categories drive the openness of bipartite genomes in *Vibrio* and *Pseudoalteromonas*. Based on this and our two recent studies, we propose a hypothesis that explains how chromids and the chromosome terminus region contribute to the genomic plasticity of bipartite genomes.

## 1. Introduction

Multipartite genomes refer to the presence of multiple replicons in a single bacterial cell and include one large chromosome, as well as one or more replicons (typically average size of 1.5 Mb), called chromids [1,2]. Bacteria with multipartite genomes are commonly found as pathogens or symbionts in animals, humans, and plants, as well as free-living bacteria [3,4] Although multipartite genomes are found throughout bacteria, 92% of those currently known are found in Proteobacteria or, using the validated name of this phylum, *Pseudomonadota* [5]). They are distributed among *Alphaproteobacteria*, *Betaproteobateria* and *Gammaproteobateria*, with 25%, 46% and 28% of multipartite bacteria found in each group, respectively [4]. Out of all multipartite bacteria, the majority (88%) are bipartite, i.e., they consist of one chromosome and one chromid.

The prevailing theory for the origin of bipartite genomes is that chromids have their origin from plasmids or megaplasmids that have been captured and domesticated by the ancestral host (the plasmid hypothesis) [1]. However, alternative hypotheses exist, such as that chromids can arise from a split of the chromosome (the schism hypothesis) [6], that the entire chromid is acquired through conjugation from another bacterium [7], or that the chromid arises through recombination between a chromid and a plasmid (chromid “rebirth”) [1]. The majority of known chromids have originated from a plasmid or megaplasmid and have plasmid-like replication machineries. For example, in *Betaproteobacteria* the majority of chromids are found within the *Burkholderiaceae* family [8] and are thought to have originated from two ancestral plasmids. Similarly, in *Alphaproteobacteria*, most chromids are found within *Rhizobiaceae* and are believed to originate from a relatively small number of plasmids [1].

Exactly why 10% of the currently available bacterial genomes are multipartite, and which purpose the extra replicons may serve is still unclear. Several hypotheses have been suggested [1,2]. One hypothesis is that chromids acquire and loose genes more rapidly, thus providing bacteria with an increased genetic plasticity. This can be advantageous in terms of environmental specialization and niche-specificity [8,9,10]. For example, studies have suggested that the gene content of chromids varies more than in chromosomes [7,11], and thus evolve more rapidly and acquire new genes at a faster rate [8], and finally, experience more relaxed selection pressure (i.e., greater evolutionary plasticity) [12]. This hypothesis is also known as the test bed hypothesis [11]. Other suggested hypotheses are that chromids can contribute with replicon-specific gene regulation and expression [13,14,15], reduce the number of overlapping replication cycles required during fast growth [16] and that extra replicons are responsible for larger genomes and increased genome content [17].

Several different calculations can be performed to provide new insights into the plasticity of multipartite genomes, and potentially differentiate between the alternative hypotheses of their existence. One commonly used approach is to estimate the rate of growth of the so-called pangenome of a species (or genus or a family), also known as the “openness” of a genome [18]. The open or closed state of a pangenome depends on the ability of the bacteria to acquire new genes, for example, through horizontal gene transfer. In an open pangenome, new genes are added to the pangenome as more genomes are sequenced or added to the analysis. In contrast, a closed pangenome approaches a constant size as more genomes are added. Heap’s law can be used to describe the pangenome size and number of new genes added for each new genome sequences and is formulated as: *n* = *kN*^γ^, where *n* is the pangenome size, N is the number of genomes used and *k* and γ are the fitting parameters. If γ < 0, the pangenome is closed, and if γ > 0, the pangenome is open [19].

Another frequently used method to study the flexibility of genomes and horizontal gene transfer, is through calculation of codon usage. Codon usage can differ between organisms, as well as between genes of the same genome [20,21]. The typical codon usage of an organism, i.e., the preferential use of certain synonymous codons in typical genes, can be distinguished from the codon usage of highly expressed genes (optimal codon usage), and codon usage of horizontally transferred genes (HTGs) (atypical codon usage) [22,23]. Optimal codon usage corresponds to the use of the most abundant tRNAs in the organism, thus leading to faster translation (protein synthesis) [20]. HTGs on the other hand have a codon usage similar to its donor organism. To what extent the codon usage of an HTG deviates from the recipient genomes depends on how distantly related the donor and recipient genomes are. Variations in relatedness between the donor and recipient, as well as amelioration (that codon usage evolves towards that of the typical genome over time) are limitations that can lead to underestimation of HTGs [24].

Within *Gammaproteobacteria*, bipartite genomes are exclusively found in *Vibrionaceae* and *Pseudoalteromonas,* both of which belong to the *Enterobacterales* order (according to the Genome Taxonomy database (GTDB)) [25]. *Vibrionaceae* consists of eight genera, all of which have bipartite genomes, whereas *Pseudoalteromonas* is the only bipartite genus among the 44 genera within *Alteromonadaceae*. According to estimates of time since divergence, *Pseudoalteromonas* is much younger than *Vibrionaceae* [26,27]. Both the *Vibrionaceae* and the *Pseudoalteromonas* chromids are believed to have originated from plasmids from the same order [26,28,29,30,31,32]. The replication of chromosomes and chromids of *Vibrionaceae* have been heavily studied, with research showing that both replicons are bidirectionally replicated, and the replication is highly coordinated with synchronized termination of the replicons [16,33,34]. Replication of most *Pseudoalteromonas* chromids occur in an unidirectionally manner, while some are replicated bidirectionally. Additionally, the replication termination has been proposed to be synchronized [27]. We recently studied the global gene distribution and gene expression in *Vibrionaceae* [35] and *Pseudoalteromonas* [32]. Briefly, we calculated the pangenomes of 124 *Vibrionaceae* and 25 *Pseudoalteromonas* genomes, mapped the pangene categories on the genomes and compared the gene distribution with gene expression under fast and slow growth conditions. In both cases, core and softcore genes were overrepresented around the origin of replication (*ori1*), whereas shell and unique genes densely populated the regions surrounding the replication terminus (*ter1*). Gene expression strongly correlated with the distance to *ori1*, with higher expression levels closer to *ori1*. The *Vibrionaceae* chromids did not display any distinct gene distribution pattern. In contrast, the core genes of *Pseudoalteromonas* chromids were found to have a strong correlation with *ter2*, regardless if the chromid was replicated bi- or uni-directionally. Gene expression in chromids did not correlate with distance to *ori* or *ter*. Based on the subcellular organization of chromosome and chromid in *Vibrio cholerae* [15,16,36,37] we found that core/softcore and shell/cloud was spatially separated into separated intracellular regions (the poles of *V. cholerae*). This led us to propose a hypothesis that the bipartite genome structure enables intracellular spatial separation of different pangene categories and that there is a connection between gene placement and gene function. 

Extensive research has been conducted on the maintenance and advantages provided by chromids in multipartite bacteria. Some hypotheses propose that chromids provide advantages such as replication specific gene regulation, increased gene content and reduced replication cycles during fast growth [13,14,15,16,17]. Other hypotheses suggest that chromids offer increased genomic plasticity and that they are a preferred location for horizontally transferred genes [8,9,11,12]. However, the extent to which chromosomes and chromids contribute to the overall plasticity and openness of bipartite genomes is not well understood. Our study aims to address this knowledge gap by calculating the openness of chromids and chromosomes of the bipartite bacteria *Vibrio* and *Pseudoalteromonas*, as well as monopartite genomes, and use codon usage and horizontal gene transfer analysis to determine which genes that contribute to the openness. Based on our data and two recent studies, we propose a hypothesis that describes how chromids and a specific region of the chromosomes appear to contribute to the genomic plasticity of bipartite genomes. Additionally, we establish the origin of *Vibrionaceae* and *Pseudoalteromonas* chromids.

## 2. Results

### 2.1. Vibrio and Pseudoalteromonas Belong to the Same Bacterial Order

**Figure 1:**
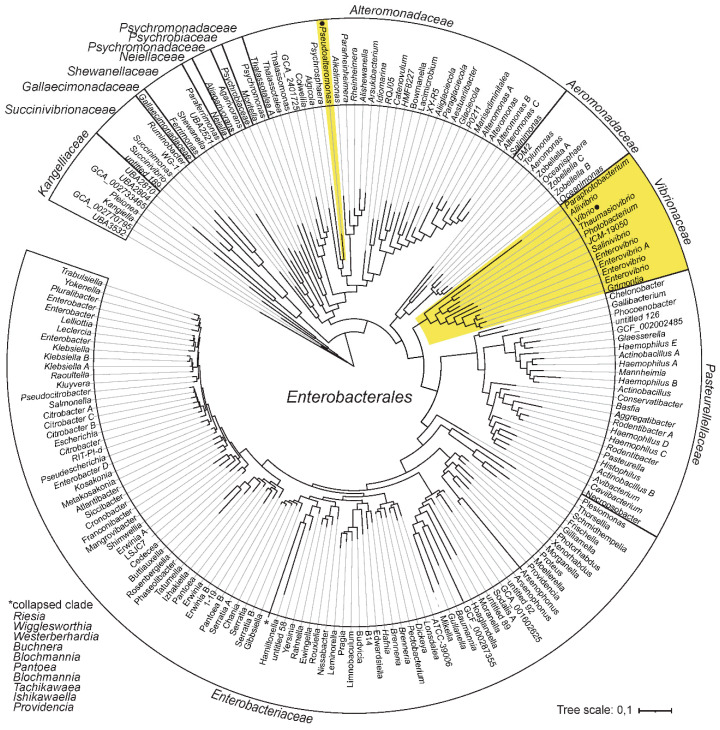
Phylogeny and distribution of bipartite genomes within *Enterobacterales*. Phylogenetic relationship between bacterial families and their respective genera are derived from the Genome Taxonomy database (GTDB). Lineages with bipartite genomes are highlighted in yellow, and genera investigated in this study are indicated with black dots.

The only known cases of bacteria with bipartite genomes within the class of *Gammaproteabacteria* are *Pseudoalteromonas* and *Vibrionaceae*. The overall phylogenetic relationship between bacterial families and their respective genera that form the order *Enterobacterales* are presented in Figure 1. The phylogenetic tree is based on information derived from GTDB release 89 [25], and lineages with bipartite genomes are highlighted.

The fact that *Vibrionaceae* and *Pseudoalteromonas* belong to the same order, raises the possibility, although unlikely, that their chromids originate from a single acquisition event in a common ancestor. Such a scenario would invoke a common origin followed by long-term retainment of the chromid, and then massive losses in all representatives of *Enterobacterales*, except *Vibrionaceae* and *Pseudoalteromonas*. A more likely explanation is that the chromids originate from two separate acquisition events.

### 2.2. Separate Origin of Chromids in Vibrionaceae and Pseudoalteromonas

We used ParA and ParB as phylogenetic markers to discriminate between the two hypotheses, i.e., a common or separate origin of the *Vibrionaceae* and *Pseudoalteromonas* chromids. ParA and ParB have fundamental roles in partitioning of replicons [38], and their conserved function and widespread distribution in bacteria and archaea make them suitable for establishing the origin of the chromids. A concatenated ParA–ParB alignment was created from sequences identified by BLASTp when using ParA and ParB sequences from *Pseudoalteromonas* and *Vibrionaceae* chromids as queries against the nr. protein database. The final dataset included a total of 376 residues from ParA and 313 residues from ParB (few residues were kept due to highly divergent regions that could not be reliably aligned).

**Figure 2:**
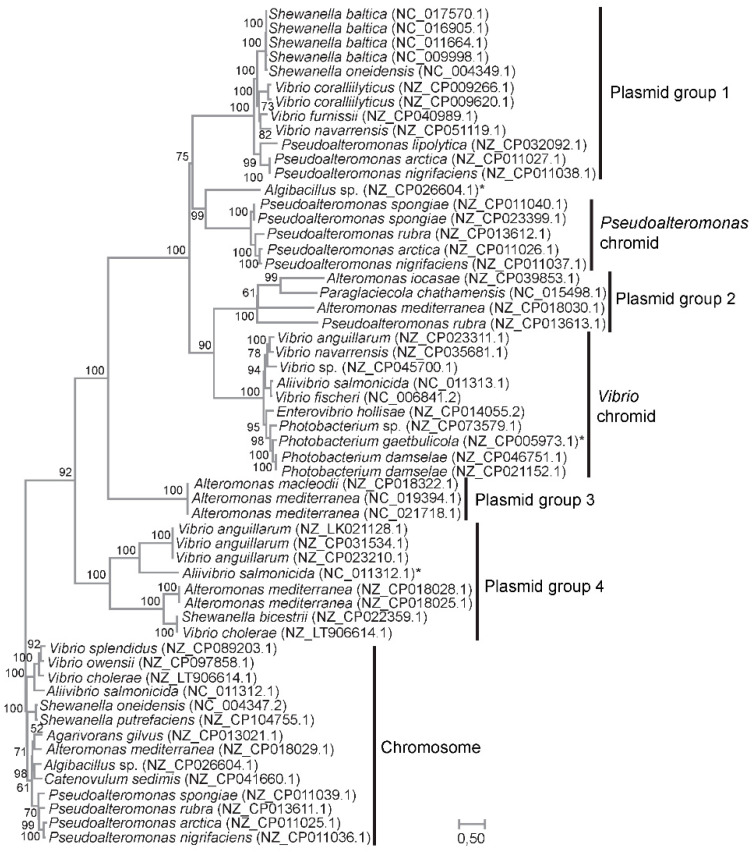
ML-tree based on the concatenated protein sequences of ParA and ParB and the WAG + G+I model. The tree shows the evolutionary relationships between chromidal sequences from *Vibrio* and *Pseudoalteromonas*, and sequences from plasmids carrying related ParA and ParB pairs. Chromosomal sequences were used as the outgroup. Clades containing plasmid sequences were designated Plasmid group 1–4 for clarity. Asterix denotes chromosomal sequences with an auxiliary pair of ParA and ParB. Bootstrap values (ML method, WAG + G+I model, 1000 pseudoreplicates) are associated with the nodes. Branch lengths are proportional to the number of substitutions per site (see scale).

The resulting maximum likelihood tree, based on the concatenated protein sequences of ParA and ParB and the WAG + G+I model, shows the evolutionary relationships between chromidal sequences from *Vibrio* and *Pseudoalteromonas* (Figure 2). Chromosomal sequences were used as the outgroup. Here, chromidal ParA–ParB from *Vibrionaceae* branches together with plasmid sequences from *Alteromonas*, *Pseudoalteromonas* and *Paraglaciecola* (Plasmid group 2), whereas chromidal *Pseudoalteromonas* ParA–ParB form a sister group with another set of plasmids, i.e., from *Shewanella, Vibrio* and *Pseudoalteromonas* (Plasmid group 1). These relationships are supported by bootstrap values of 90% and 75%, respectively. In summary, our result agrees with separate origins of the *Vibrionaceae* and *Pseudoalteromonas* chromids and suggests that both chromids were acquired from plasmids belonging to the *Enterobacterales* gene pool. 

### 2.3. The Chromids in Pseudoalteromonas and Vibrio Play a Significant Role in the Openness of the Two Genomes

It has been proposed that the main advantage of keeping multiple replicons is increased genetic flexibility, often termed “openness” (e.g., [8,11,12,32]). A commonly used method to estimate the openness of a pangenome, is to perform curve fitting of the pangenome size versus number of genomes using Heaps’ law [18,19]. Heaps’ law is formulated as *n* = *kN*^γ^, where an exponent γ > 0 indicates an open pangenome, i.e., the pangenome will grow/gain genes as new genomes are sequenced and added to the analysis. An exponent γ < 0 indicates a closed pangenome that will not grow in size as new genomes are added. To estimate to what extent the chromosome and the chromid contribute to the pangenome openness we made two separate datasets consisting of 50 complete *Vibrio* and 26 complete *Pseudoalteromonas* genomes. The datasets are non-redundant, meaning that only one complete genome per available species was included (see Table S1 for complete list of bipartite genomes). We then calculated the pangenome size and Heaps’ exponent for the chromosome, chromid and total genome (see Table S3). The pangenome of *Vibrio* consists of 822 core (encoded by all 50 genomes), 1505 softcore (encoded by ≥47 genomes), 8463 shell (encoded by ≤46 and ≥3 genomes), and 37,177 cloud (encoded by ≤2 genomes). The *Pseudoalteromonas* pangenome consists of 1386 core (encoded by all 26 genomes), 1787 softcore (encoded by ≥24 genomes), 5096 shell (encoded by ≤23 and ≥3 genomes), and finally 20,635 cloud (encoded by ≤2 genomes).

**Figure 3:**
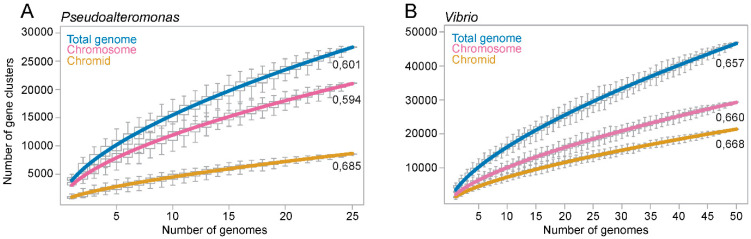
Graphs showing the calculated pangenome sizes of *Pseudoalteromonas* and *Vibrio* relative to the number of added genomes. For *Pseudoalteromonas* (**A**) and *Vibrio* (**B**), the number of gene clusters continues to grow as more genomes are added to the analysis, which shows that the chromids, chromosomes and total genomes are open. Each data point in the graph is based on the median of pangenome size of 100 randomly generated datasets (strain orders). The Heaps’ exponents are shown associated with each graph and are used to evaluate the openness of the genomes.

The calculated pangenome sizes are presented (Figure 3), with the sizes being relative to the number of genomes added (median of 100 randomly generated combinations of genome datasets). For both *Vibrio* and *Pseudoalteromonas*, the size of the chromosomal, chromidal and total genomes increase as more genomes are added to the analysis, more in the beginning of the curve and less after 10 genomes are added. The Heaps’ exponent associated with the *Vibrio* chromid (0.668 ± 0.001) and the chromosome (0.660 ± 0.003) are virtually identical. This means that the two replicons are equally “open”, but because of its bigger size, the chromosome hosts the majority of new genes. For *Pseudoalteromonas*, the chromid exponent (0.685 ± 0.007) is considerably larger than that of the chromosome (0.594 ± 0.002) and total genome (0.601 ± 0.003). With the highest Heaps’ exponent, the chromid contributes considerably to the openness of the *Pseudoalteromonas* genome. In summary, we have used Heaps’ law to evaluate the openness of the chromosome and chromid of *Vibrio* and *Pseudoalteromonas* by calculating the pangenome sizes and Heaps’ exponents. The *Vibrio* chromosome and chromid are equally open, whereas the *Pseudoalteromonas* chromid is more open than the chromosome.

### 2.4. Bipartite Genomes Are More Open Compared to Monopartite Genomes

Next, we compared the openness of the *Pseudoalteromonas* and *Vibrio* genomes to that of monopartite genomes of closely related genera. Hypothetically, the structural organization of genomes into one or multiple replicons can have a major impact on the flexibility of the genomes. The four relatively closely related genera *Alteromonas, Idiomarina, Rodentibacter* and *Yersinia* (all from *Enterobacterales*) with monopartite genomes were chosen for the analysis, for comparison to bipartite genomes (see Table S2 for complete list of monopartite genomes). For each genera, the Heaps’ exponent was calculated from a random combination of an increasing number of genomes (using seven permutations) (see Table S3). This was conducted to test what effect the number of genomes and genome combinations have on the resulting Heaps’ exponent. A dataset consisting of 27 *Escherichia coli* (species level) genomes was added as a control.

**Figure 4:**
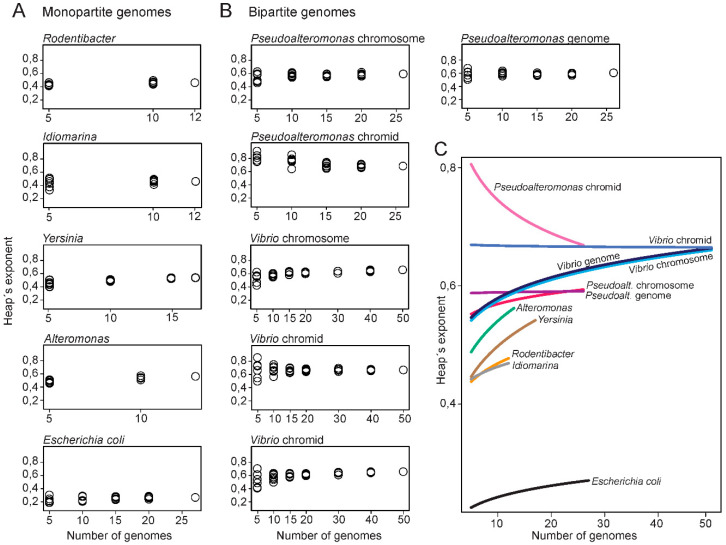
Plots of Heaps’ exponents against the number of genomes. The analysis was carried out for datasets with monopartite (**A**) or (**B**) bipartite genomes. Each of the Heaps’ exponents are made from the median number of pangenome sizes from 100 randomly generated strain orders. (**C**) Rarefaction curves of Heaps’ exponents plotted against number of genomes. The curves can be regarded as a summary and of the results from (**A**,**B**) through curve fitting of the Heaps’ exponents.

Plots with Heaps’ exponent relative to the number of genomes for monopartite genomes are presented in Figure 4A. When the number of genomes is small, the distribution of Heaps’ exponent is wide for *Yersinia, Alteromonas* and *Rodentibacter*, whereas for *Idiomarina*, the distribution is smaller. The corresponding plots for *Vibrio* and *Pseudoalteromonas,* show that the Heaps’ exponent is widely distributed when only a few numbers of genomes are included in the datasets (Figure 4B). As the number of genomes increases, the exponents are less distributed (see Table S3 for complete list of Heaps’ exponents). Similarly, the calculations for *Pseudoalteromonas* chromids vary greatly for small datasets but become more stable as the number of included genomes increases. These results show, as expected, that larger dataset (>10 genomes) result in more stable Heaps’ values.

A summary of the results from Figure 4A,B through curve fitting of the Heaps’ exponents, show that all bipartite replicons have larger Heaps’ exponents compared to the monopartite genomes (Figure 4C). For example, at 10 genome datasets the lowest Heaps’ value for bipartite are 0.618, whereas the highest Heaps’ value for monopartite are 0.572. These results show that, with the currently available genomes, bipartite genomes have more open pangenomes, and thus appear more genetically flexible than monopartite counterparts. Chromids have the most open state of all replicons compared. Notably, how the exponent will change when more genomes become available is however unclear.

In summary, we plotted the Heaps’ exponent relative to the size of genome datasets to compare openness of monopartite versus bipartite genomes. With the currently available datasets, bipartite genomes appear more open than that of closely related monopartite bacteria. 

### 2.5. Codon Usage Is Specific for Each Pangene Category Rather Than for Each Replicon Type

Next, we used codon usage bias calculations to further explore the plasticity of bipartite genomes. Newly acquired genes are expected, in general, to have different codon usage profiles compared to those of most genes, especially genes with essential cellular roles (e.g., for cellular growth). Codon bias analyses are therefore used for exploring evolutionary aspects, including lateral transfer of genes.

Therefore, we first measured the relative synonymous codon usage (RSCU) for all individual genes in each of the 50 *Vibrio* and 26 *Pseudoalteromonas* genomes and performed a correspondence analysis of the RSCU values. Variations in codon usage among different pangene categories were explored by dividing the gene datasets into core, softcore, shell and cloud genes, and visualize the gene categories in different colors. Axis1 and Axis2 correlate with the two main influencing factors of codon usage bias. They represent 10.98% and 8.07% of the total variation for *Vibrio* and 10.97% and 7.52% of the total variation for *Pseudoalteromonas*, respectively.

**Figure 5:**
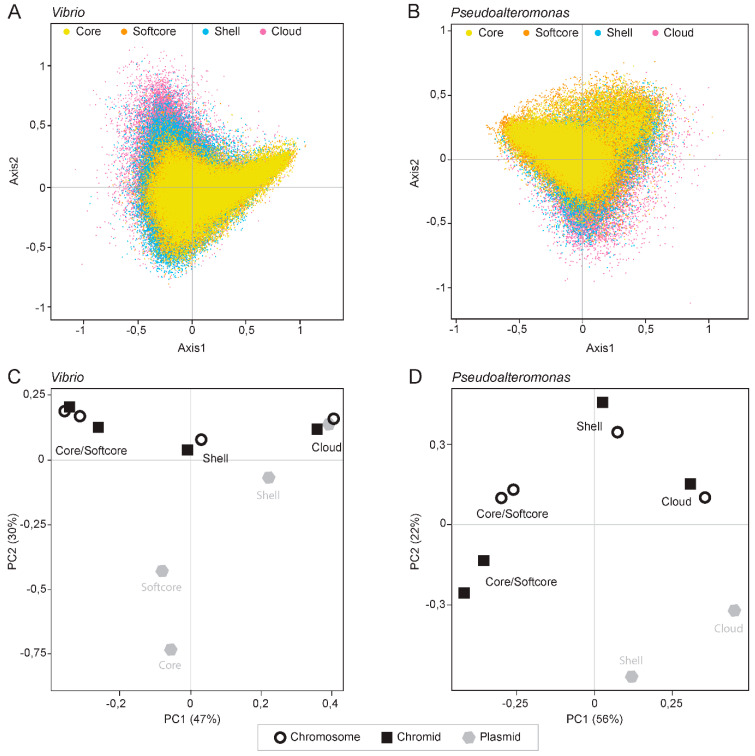
Correspondence analysis of relative synonymous codon usage (RSCU). The analyses are based on 50 *Vibrio* (**A**) and 26 *Pseudoalteromonas* (**B**) genomes. Core, softcore, shell and cloud genes are indicated with yellow, orange, blue and pink colors, respectively. The genes are distributed on primary and secondary axes which account for 10.98% and 8.07% in *Vibrio* and 10.97% and 7.52% *Pseudoalteromonas* of the total variation. Principal component analysis PCA) plots of the RSCU data from *Vibrio* (**C**) and *Pseudoalteromonas* (**D**) are shown. Both type of plots show that codon usage is specific for each pangene category rather than type of replicon.

Both *Vibrio* and *Pseudoalteromonas* have a broad distribution of codon usage, that are to a great extent specific for each pangene category (Figure 5A,B). In *Vibrio*, core and softcore genes are densely clustered toward the upper and lower right quadrants, whereas the shell and especially cloud genes are distributed towards upper left quadrant. In *Pseudoalteromonas*, core and softcore genes are distributed densely in upper left quadrant, shell genes toward the lower quadrants and in upper left quadrant.

PCA plots of the RSCU data described above (from Figure 5A,B) show that codon usage clusters based on pangene categories and not on the type of replicon (Figure 4C). This result is supported by correlation analysis of the RSCU values for each pangene category and analysis of median effective number of codons (ENC) for each pangene category (see Table S4 for global RSCU values and Table S5 for correlation plot and ENC values).

In summary, we performed COA and PCA on RSCU values to identify major trends of codon usage patterns in *Vibrio* and *Pseudoalteromonas*. Both type of plots show that codon usage is specific for each pangene category rather than type of replicon. This is valid for both *Pseudoalteromonas* and *Vibrio.* Similar codon usage for each pangene category indicates that they also have different evolutionary trajectories, which we explore further (see below). 

### 2.6. Shewanella Represents the Top Donor of HTGs to Vibrio and Pseudoalteromonas

To identify putatively horizontally transferred genes (HTGs) in *Vibrio* and *Pseudoalteromonas*, we used HGTector [39], which is a software for genome-wide detection of horizontal gene transfer events based on homology searches. For *Pseudoalteromonas*, we defined horizontally transferred genes as all genes that originate from a donor outside of *Alteromonadaceae*, whereas for *Vibrio* horizontally transferred genes come from outside *Vibrionaceae*.

**Figure 6:**
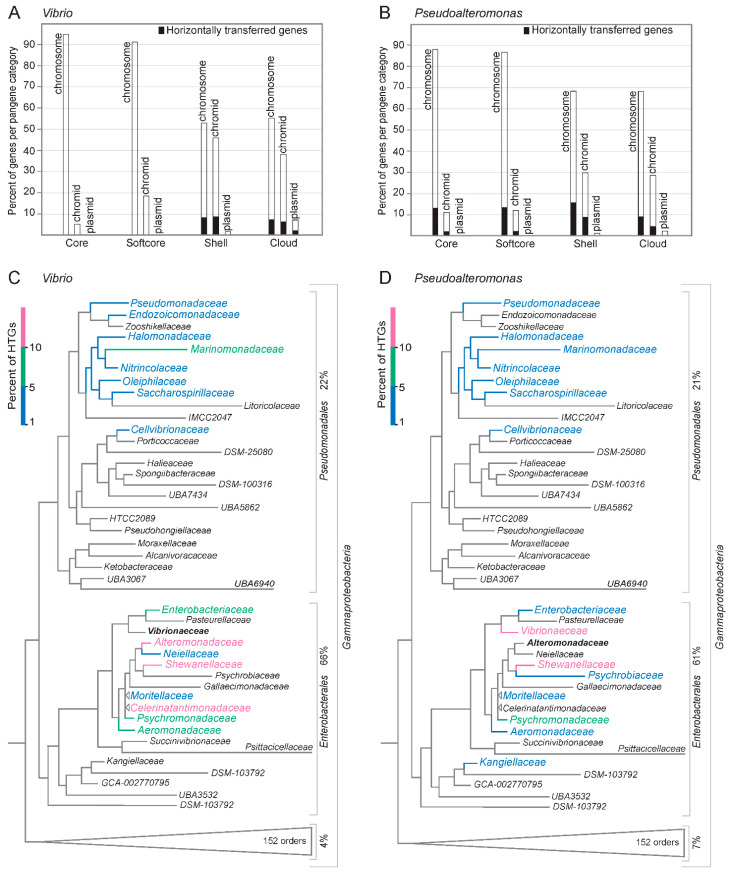
Horizontally transferred genes in *Vibrio* and *Pseudoalteromonas,* and the phylogenetic distribution of their donors. The number of HTGs in *Vibrio* (**A**) and *Pseudoalteromonas* (**B**) were predicted using the HGTector software. The data is shown as percentage of HTGs in each pangene category (core, softcore, shell and cloud), and also they are distributed among the three types of replicons (chromosomes, chromids and plasmids). HTGs were defined as genes with closest BLASTp hits outside of its family (i.e., *Vibrionaceae* and *Alteromonadaceae*, respectively). Next, the predicted bacterial donors of HTGs that reside in *Vibrio* (**C**) and *Pseudoalteromonas* (**D**) are shown mapped onto a phylogeny of *Gammaproteobacteria*. The top donors are shown in colorblindness-friendly color codes, from 1–5% (blue), 5–10% (green) and 10–15% (reddish purple). The majority of HTGs originates from other families within *Enterobacterales*, with *Shewanella* (at genus level) as the top donor to both *Vibrio* and *Pseudoalteromonas*.

The number of HTGs detected for each pangene category on each replicon is presented in Figure 6A,B. HTGs comprise 11% and 23% of the total number of genes in the pangenomes in *Vibrio* [24,529 genes/7308 gene clusters (12 core, 32 softcore, 1496 shell, 4765 cloud)] and *Pseudoalteromonas* [19,970 genes/4310 gene clusters (309 core, 424 softcore, 2510 shell, 2389 cloud)], respectively. In *Vibrio,* the majority of HTGs (98%) are shell or cloud genes. These are distributed on the chromosome, where they make up 15% of shell and 13% of cloud genes, and on the chromid where they make up 20% (shell) and 16% (cloud). Notably, the *Vibrio* dataset contains 35 plasmids (from 19 genomes), of which 27% of shell genes and 13% of cloud genes are HTGs. For *Pseudoalteromonas*, about half of the HTGs are core and softcore genes. Of these, 15% and 18% of softcore genes are distributed on chromosomes and chromids, respectively. The other half of HTGs corresponds to chromosomal genes where they make up 24% of shell and 12% of cloud genes, respectively, and the corresponding numbers for chromidal genes are 30% (shell) and 13% (cloud). Six genomes contain one plasmid each. Here, 30% of HTGs represent shell and 14% represent cloud genes.

To summarize, in *Vibrio*, the identified horizontally transferred genes are typically shell and cloud genes located on both the chromosomes and chromids. In *Pseudoalteromonas,* the HTGs are more evenly distributed among all pangene categories from both chromosomes and chromids.

Phylogenetic distribution of the bacterial gene donors, i.e., the bacterial families from where the predicted HTGs originated from, show that in both *Vibrio* and *Pseudoalteromonas* the main contributors are families within the *Gammaproteobacteria* orders *Enterobacterales* and *Pseudomonadales* (Figure 6C,D). *Enterobacterales* and *Pseudomonadales* accounts for 66% and 22% of the total HTGs in *Vibrio* and 61% and 21% in *Pseudoalteromonas*, respectively. For *Pseudoalteromonas*, the top three donor genera are *Shewanella* (17%; *Shewanellaceae*), followed by *Vibrio* (11%; *Vibrionaceae*) and *Photobacterium* (5%; *Vibrionaceae*). Similarly, for *Vibrio* the top three donors are *Shewanella* (13%; *Shewanellaceae*), *Marimonas* (6%; *Marinomonadaceae*), and *Psychromonas* (6%; *Psychromonadaceae*).

In summary, we found that the majority of HTGs in *Vibrio* and *Pseudoalteromonas* originates from *Enterobacterales* and *Pseudomonadales*, with *Shewanella* representing the top donor of all genera. 

## 3. Discussion

Here, we continue our studies on the bipartite genomes of *Vibrionaceae* and *Pseudoalteromonas*. According to GTDB, *Vibrionaceae* and *Pseudoalteromonas* both belong to *Enterobacterales* [25]. Based on an inferred ParAB phylogeny, we first established that the *Vibrio* and *Pseudoalteromonas* chromids do not share the same last common ancestor. The chromids originate from two separate plasmid acquisition events from plasmids within the *Enterobacterales* gene pool. We then calculated the pangenome and openness of the *Vibrio* and *Pseudoalteromonas* genomes and found that the *Vibrio* chromosome and chromid are equally open (i.e., the chromosome and chromid pangenome size increase at a similar rate as more genomes are added to the analysis), whereas the *Pseudoalteromonas* chromid is more open than the chromosome. Compared with monopartite genomes, bipartite are more open, at least based on today’s available genome datasets. We next used codon usage bias calculations to elucidate which type of genes are more likely to have been acquired horizontally, thus leading to open bipartite genomes in *Vibrio* and *Pseudoalteromonas.* The data support that codon usage is specific to each pangene category regardless of which replicon they reside in. The vast majority of HTGs in *Vibrio* are shell or cloud genes, whereas HTGs in *Pseudoalteromonas* are more evenly distributed among all pangene categories. 

By comparing the bipartite genomes of *Vibrio* and *Pseudoalteromonas* with monopartite genomes of related bacterial families, we showed that bipartite genomes appear more open than monopartite. The increased openness suggests that bipartite genomes have a higher capacity to acquire genes [40]. Using codon usage bias calculations and the HGTector tool we, therefore, set out to identify which type of genes are typically horizontally acquired by vibrios and pseudoalteromonases. We found that the codon usage in both *Vibrio* and *Pseudoalteromonas* group based on which pangene category genes belong to, and not based on which replicon genes reside on (chromidal or chromosomal placement). Notably, codon usage of cloud genes differs most from that of core genes (compared to shell genes), which are typically more highly expressed and therefore assumed to use codons better adapted to the translation machinery (adaption) [18,21]. This supports that cloud genes include a higher portion of more recently acquired genes. A similar pattern was reported for the multipartite bacterium *Sinorhizobium meliloti*, where codon usage of core genes on the chromosome and chromid were more similar than when compared to unique genes on the same replicons [41]. To conclude, less optimal codon usage of shell and cloud genes agree with data from our HGTector analysis, which suggests that as much as 98% of the detected HTGs in vibrios are either cloud or shell genes.

For *Pseudoalteromonas*, the general picture is similar, but here the HGTector result suggests that about half of the HTGs are core/softcore genes, whereas the other half corresponds to shell and cloud genes. The high proposition of HTGs among core/softcore is somewhat puzzling to us. To be detected as HTG, BLAST searches must identify the closest hit outside of *Alteromonadaceae*. We speculate that this result can be explained by the fact that *Pseudoalteromonas* is relatively young compared to *Vibrio* [502–378 vs. 1100–900 million years ago [26,27], respectively], and more genes will thus potentially be identified as HTG among core/softcore. The rationale is that HTGs in the last common ancestor (LCA) of extant *Pseudoalteromonas* bacteria have had approx. 500 million fewer years to adapt to the translation machinery than the corresponding genes in *Vibrio*. Moreover, *Pseudoalteromonas* have had less time to diverge from the LCA into different species, which subsequently can occupy various biological niches (such as *Vibrio*, that comprises at least 140 species). Consequently, our pangenome analyses identified 1386/1787 and 822/1505 core/softcore genes in *Pseudoalteromonas* and *Vibrio*, respectively. To summarize, HTGs in *Vibrio* are almost exclusively from the shell and cloud categories, whereas about half of HTGs in *Pseudoalteromonas* are shell and cloud genes.

**Figure 7:**
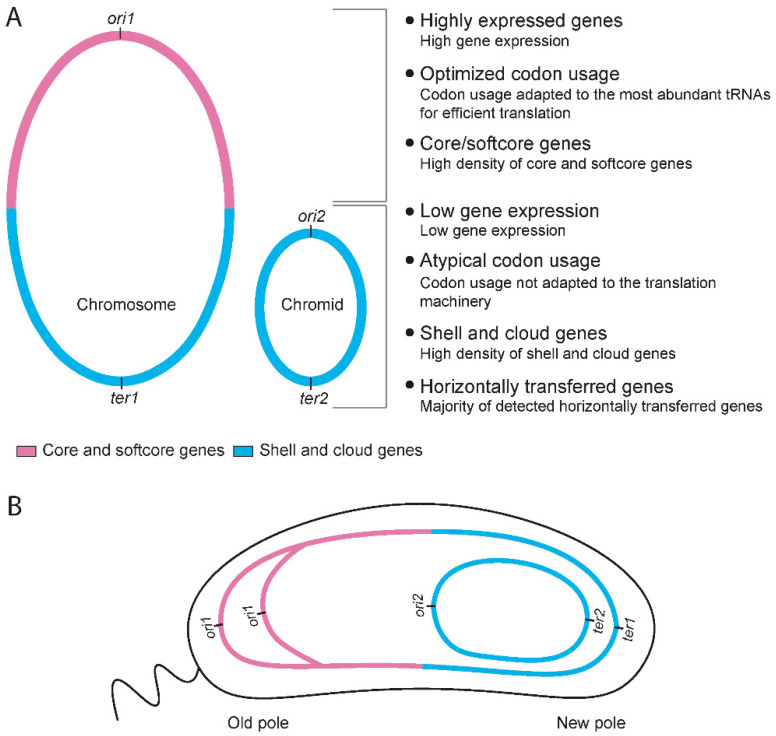
Summary of key characteristics of bipartite genomes in *Vibrio* and *Pseudoalteromonas*, and a putative model for accepted landing sites of HTGs. (**A**) Genes on the upper half of the chromosome are statistically more highly expressed, more likely to be core or softcore genes, and the codon usage is well adapted to the translational machinery. Genes located on the lower half of the chromosome, or the chromid, are statistically lower expressed, more likely to be shell or cloud genes, and have atypical codon usage less adapted to the translational machinery (compared to core/softcore). (**B**) Sketch of a hypothetical cell with a bipartite genome, and depicting the subcellular location of a chromosome and a chromid. The model is based on our pangenome calculations and genomic mapping of pangene types [32,35], and data from *V. cholerae* where the subcellular position of replicons have been determined [15,16,36,37]. Based on the genomic characteristics described in A, we hypothesize that chromids and the lower halves of the chromosomes are favored “landing sites” for gene acquisition in bipartite genomes.

Based on the results presented above, a new question arises: if a significant portion (>98% and >50%) of HTGs belong to the shell and cloud categories, where in the genomes are they typically located, and could their location explain why bipartite genomes are more flexible than monopartite genomes? In the light of this and previous studies, we suggest that the chromid and the lower half of the chromosome are particularly available for integration of new genes, and thus contribute to the elevated flexibility/openness of bipartite genomes (Figure 7). We recently mapped the pangene categories on the genomes of *Vibrionaceae* [35] and *Pseudoalteromonas* [32] and discovered distinct distribution patterns. On the chromosomes, core and softcore genes are overrepresented around the origin of replication (*ori1*), whereas shell and unique genes densely populate the regions surrounding the replication terminus (*ter1*). The *Vibrionaceae* chromids showed no clear gene distribution pattern, but for *Pseudoalteromonas*, the distribution of core genes strongly correlates with *ter2*, regardless of its position [i.e., *Pseudoalteromonas* chromids are replicated bi- or uni-directional, hence the position of *ter2* varies [27]]. Other studies have also found a correlation between density of mobile genetic elements and proximity to the *ter* region. Kopetja et al., discovered that in *Rhodobacterales*, core genes are located near *oriC*, whereas phages are located near the terminus [42]. A similar finding was reported by Oliviera et al. [43]. Using a diverse genome dataset, they found a higher frequency of “hot-spots” for horizontal gene transfer that contained prophages near *terC*. The evolutionary process responsible for this distribution pattern is discussed elsewhere [32,35], but from the current results we conclude that chromids and the lower halves of chromosomes appear to be favored “landing sites” for gene acquisition in bipartite genomes.

## 4. Material and methods

### 4.1. Enterobacterales Reference Tree

The phylogenetic tree of *Enterobacterales* was made using Annotree [44], which is based on phylogeny and taxonomic nomenclature from the Genome Taxonomy database (GTDB) [25]. According to GTDB, *Pseudoalteromonas* and *Vibrionaceae* both group within the order *Enterobacterales*. Whereas following the NCBI taxonomy classification, *Vibrionaceae* and *Pseudoalteromonas* belong to separate orders (i.e., “*Vibrionales*” and *Pseudoalteromonadales*). Notably, in addition to multipartite genomes in *Vibrionaceae* and *Pseudoalteromonas*, there are reports of single strains with chromids in *Alteromonas mediterranea* [45] and in *Plesiomonas shigella* [46].

### 4.2. ParAB phylogenetic tree

BLASTp was used to compile ParA and ParB protein sequences from the databases using ParA and ParB from *Vibrionaceae* and *Pseudoalteromonas* as queries. The protein sequences were aligned using MUSCLE [47]. The alignment was manually adjusted using BioEdit [48], and only unambiguously aligned positions were kept for phylogenetic inference. A total of 689 aa positions were kept. MEGA11 was used to generate a Maximum Likelihood (ML) tree using the WAG model, Gamma distribution of evolutionary rates among sites, with invariant sites allowed (WAG + G + I) [49,50]. Bootstrap analysis with the same parameters as described above was performed with 1000 pseudoreplicates.

### 4.3. Genome Retrieval and Gene Annotation

One dataset for each of the genera *Pseudoalteromonas, Vibrio, Alteromonas, Yersinia*, *Idiomarina* and *Rodentobacter* and *E. coli* was made based on taxonomy of Genome Taxonomy database [25]. The genomes were downloaded from the RefSeq database at National Center for Biotechnology Information (NCBI) [51]. All *Vibrio* and *Pseudoalteromonas* genomes were complete (see Table S1 for complete lists of bipartite genomes). For a bipartite genome to be included in the study, its chromid had to meet the following criteria: it must possess a plasmid-type replication system, have a nucleotide composition close to that of the chromosome and contain core genes [1]. Direct evidence of the physical presence of chromids exist for *V. cholerae* [15,16,52]. and in *Pseudoalteromonas tunicata* and *Pseudoalteromonas spongiae* [27], all of which are included in the study. We allowed draft genomes with up to 200 contigs to be included for datasets of monopartite genomes (*Alteromonas, Yersinia*, *Idiomarina* and *Rodentobacter* and *E. coli*) (see Table S2 for complete list of monopartite genomes). All genomes were re-annotated using RAST (Rapid Annotation using Subsystem Technology) version 2.0 [53]. To make the datasets non-redundant, FastANI [54] was used to calculate average nucleotide identity values for all genomes against all genomes to select one genome per species.

### 4.4. Pangenome Calculation

To classify the annotated protein sequences of each of the seven datasets from *Pseudoalteromonas, Vibrio, Alteromonas, Yersinia, Idiomarina, Rodentobacter* and *E. coli* into four pangenome categories, we performed pangenome analysis using the clustering algorithm MCL in the software package GET_HOMOLOGUES ( https://github.com/eead-csic-compbio/get_homologues, accessed on 15 August 2022)) [55]. The parameter “minimum percent sequence identity” was set to 50 and “minimum percent coverage in BLAST query/subj pairs” was set to 75 (default) [56]. To calculate the openness of pangenomes, pangenome analysis was performed using 100 permutations (for each datapoint). The median values of the combinations was used to perform curve fitting and calculate Heaps’ exponent using power-law regression in the “aomisc package” in R v.4.0.3 [57] (see Table S3).

### 4.5. Calculation of Codon Usage

To investigate codon usage bias, codonW [58] was used to calculate relative synonymous codon usage (RSCU) and perform correspondence analysis of all genes in *Pseudoalteromonas* and *Vibrio.* Correspondence analysis (COA) was used to identify the major trends of codon usage among the four pangene categories. Each gene is described by a vector of 59 variables (codons) that correspond to the RSCU value of each synonymous codon. Codons without synonymous alternatives were excluded from the analysis (methionine, tryptophane and stop codons UAA, UAG, UGA). CodonW was also used to calculate global RSCU values of the pangenome categories separated based on their respective replicon (either chromosome, chromid or plasmid). The RSCU values were then plotted on a principal component analysis (PCA) (see Table S4 for global RSCU values). Effective number of codons was calculated using the R package “vhcub” [59] (see Table S5). ENC is used to estimate the overall codon bias for each gene in a dataset. ENC values range from 20 to 61, where all synonymous codons are used equally at 61 and only one codon used at 20 [60].

### 4.6. Prediction of Horizontally Transferred Genes

HGTector v2.0b3 [39] was used to identify putatively horizontally transferred genes in *Vibrio* and *Pseudoalteromonas*. A database consisting of 25,859 bacterial RefSeq proteins was downloaded from NCBI [51] and compiled using DIAMOND [61]. DIAMOND BLASTP searches with *Vibrio* pangenes and *Pseudoalteromonas* pangenes as queries was performed with the parameters e-value < 1 × 10^−5^, sequence identity > 30%, and sequence coverage > 50%. To search for horizontally transferred genes in *Pseudoalteromonas*, the parameter “self group” was set to *Pseudoalteromonas* (TaxID: 53246) and “close group” to *Alteromonadaceae* (TaxID: 226, 2848171, 135575, 28228, 1621534, 2071980, 336830, 2800384, 67575, 89404, 1249554, 111142, 2800384, 907197, 1518149, 366580, 1751872, 249523, 265980, 1407056, 2834759, 2125985, 296014, 1406885, 1172191, 137583, 2848177, 2661818, 2798470, 2851088). To search for horizontally transferred genes in *Vibrio*, the parameter “self group” was set to *Vibrio* (TaxID: 662) and “close group” was set to *Vibrionaceae* (TaxID: 641).

### 4.7. Statistical Analysis

Statistical analysis was performed using R in RStudio [62]. Correlation analysis was performed using the cor() function with Pearsons correlation.

## Data Availability

The data presented in this study are available in Table S1 and Table S2.

## Supplementary Materials

The following supporting information can be downloaded at: https://www.mdpi.com/article/10.3390/ijms24054292/s1.
